# ESCRTs regulate amyloid precursor protein sorting in multivesicular bodies and intracellular amyloid-β accumulation

**DOI:** 10.1242/jcs.170233

**Published:** 2015-07-15

**Authors:** James R. Edgar, Katarina Willén, Gunnar K. Gouras, Clare E. Futter

**Affiliations:** 1 Department of Cell Biology, UCL Institute of Ophthalmology, London EC1 V9EL, UK; 2 Cambridge Institute for Medical Research, University of Cambridge, Cambridge CB2 0XY, UK; 3 Department of Experimental Medical Science, Lund University, Lund 22184, Sweden

**Keywords:** Amyloid precursor protein, Multivesicular body, Alzheimer's disease, ESCRT

## Abstract

Intracellular amyloid-β (Aβ) accumulation is a key feature of early Alzheimer's disease and precedes the appearance of Aβ in extracellular plaques. Aβ is generated through proteolytic processing of amyloid precursor protein (APP), but the intracellular site of Aβ production is unclear. APP has been localized to multivesicular bodies (MVBs) where sorting of APP onto intraluminal vesicles (ILVs) could promote amyloidogenic processing, or reduce Aβ production or accumulation by sorting APP and processing products to lysosomes for degradation. Here, we show that APP localizes to the ILVs of a subset of MVBs that also traffic EGF receptor (EGFR), and that it is delivered to lysosomes for degradation. Depletion of the endosomal sorting complexes required for transport (ESCRT) components, Hrs (also known as Hgs) or Tsg101, inhibited targeting of APP to ILVs and the subsequent delivery to lysosomes, and led to increased intracellular Aβ accumulation. This was accompanied by dramatically decreased Aβ secretion. Thus, the early ESCRT machinery has a dual role in limiting intracellular Aβ accumulation through targeting of APP and processing products to the lysosome for degradation, and promoting Aβ secretion.

## Abstract (precis)

**Highlighted Article:** The early ESCRT machinery has a dual role in limiting intracellular Aβ accumulation through targeting of APP and its processing products to lysosomes for degradation, and promoting Aβ secretion.

## INTRODUCTION

Alzheimer's disease is characterized by progressive loss of memory and cognitive function, and histologically characterized by neuronal loss, extracellular amyloid plaques, dystrophic neurites and neurofibrillary tangles of hyperphosphorylated Tau (Glenner, 1989; Goedert et al., 1988; Selkoe et al., 1986). Plaques are extracellular aggregates of Aβ peptides (Glenner and Wong, 1984) generated by sequential proteolysis of APP by β-site APP cleaving enzyme 1 (BACE1) and the γ-secretase complex. APP can alternatively be processed by α-secretase, which cleaves within the Aβ sequence, precluding Aβ formation.

Both the trans-Golgi network (TGN) and the endolysosomal pathway contain APP, BACE1 and γ-secretase, and are proposed sites of Aβ generation. Newly synthesized APP can traffic from the TGN to endosomes either directly through binding to the adaptor AP-4 (Burgos et al., 2010) or via the plasma membrane, and can also undergo retromer-dependent recycling to the TGN (Vieira et al., 2010). APP and its processing products have been localized to multivesicular bodies (MVBs) in Alzheimer's disease brains (Takahashi et al., 2004, 2002) and cultured neurons (Morel et al., 2013). MVBs have a number of fates including fusion with the lysosome, fusion with the cell surface for the release of intraluminal vesicles (ILVs) as exosomes, fusion with autophagosomes to generate amphisomes and the biogenesis of lysosome-related organelles such as melanosomes. In pigmented cells PMEL is targeted to ILVs where it undergoes proteolytic processing to generate amyloid striations upon which melanin is deposited (Berson et al., 2001).

Several populations of MVBs exist (White et al., 2006) and ILVs can be formed by different mechanisms (Edgar et al., 2014; Stuffers et al., 2009; Subra et al., 2007; Trajkovic et al., 2008; Wollert and Hurley, 2010). The endosomal sorting complexes required for transport (ESCRT) machinery, which is composed of four complexes (0–III), binds ubiquitylated cargo and generates ILVs that are delivered to lysosomes and degraded. APP can be ubiquitylated (Watanabe et al., 2012) in a manner that promotes its targeting to ILVs (Morel et al., 2013). Interfering with the potential interaction between early ESCRT components and APP has been reported to both increase (Morel et al., 2013) and decrease (Choy et al., 2012) Aβ secretion. Depleting later ESCRT components promotes APP traffic to the TGN and increased Aβ secretion (Choy et al., 2012). Intriguingly PMEL sorting onto ILVs is essential for its amyloidogenic processing but occurs independently of ESCRTs (Theos et al., 2006). The extent to which ESCRT-dependent targeting of APP to ILVs might play a similar positive role in Aβ production, or might reduce Aβ production by targeting APP to the lysosome and/or removing APP from the recycling pathway to the TGN, remains unclear.

Studies of the role of the ESCRT machinery in regulating APP traffic and Aβ production have been performed in a variety of cultured cell lines and primary neurons, in which intracellular Aβ is difficult to detect. Most studies have therefore analysed ESCRT roles in Aβ secretion, rather than intracellular accumulation. The importance of intracellular versus extracellular Aβ is a subject of considerable debate. Transgenic mice display intraneuronal Aβ at the same age as initial pathological manifestations and synaptic dysfunction, which is prior to the appearance of extracellular plaques (Oddo et al., 2003). In this study, we investigate the role of ESCRT-mediated sorting within MVBs in intracellular Aβ accumulation. We reveal a role for early ESCRT components in limiting intracellular Aβ accumulation by both promoting lysosomal targeting and promoting Aβ secretion.

## RESULTS

### APP is localized to the Golgi and ILVs of a subpopulation of MVBs in H4-APP cells

**Fig. 1.**
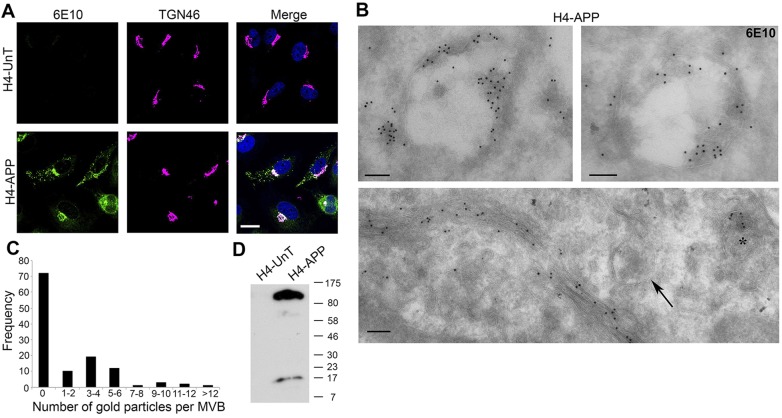
**APP localizes to the Golgi and ILVs of a subpopulation of MVBs.** (A) Untransfected H4-UnT and H4-APP cells were stained for APP/Aβ (6E10; green), TGN46 (magenta) and DAPI (blue). Scale bar: 10 μm. (B) In ultrathin cryosections of H4-APP cells, 6E10 immunogold labelling is on a subset of MVBs (asterisks) and the Golgi, although some MVBs lack label (arrows). Scale bars: 100 nm. (C) Counting 6E10 gold particles per MVB (*n*=120) shows populations of 6E10 negative and positive MVBs (approximately four particles per MVB). (D) H4-UnT and H4-APP cells were western blotted with 6E10.

Immunofluorescent staining of H4 neuroglioma cells stably expressing human APP (H4-APP) with the antibody 6E10, which labels both APP and Aβ (hereafter anti-APP/Aβ antibody), revealed that there was a perinuclear pool that colocalized with the Golgi marker TGN46 (also known as TGOLN2), plus punctate staining, typical of endosomes and lysosomes (Fig. 1A). As no marker accurately distinguishes between MVBs and lysosomes, electron microscopy was used to determine the identity of the punctate staining. We previously showed that electron lucence, a diameter >200 nm, and one or more discrete ILVs in a single section plane defines MVBs that are functionally distinct from lysosomes, as they lack the capacity to degrade endocytosed EGF (Futter et al., 1996; Wherlock et al., 2004). Lysosomes are electron dense, can contain ILVs but also contain membrane whorls and can degrade endocytosed EGF (Futter et al., 1996; Wherlock et al., 2004). Immunoelectron microscopy showed that the majority of 6E10 staining was confined to the Golgi and MVBs, where most was on ILVs but some was on the limiting membrane (Fig. 1B). Analysis of the 6E10 gold distribution on MVBs revealed the labelling not to be randomly distributed but, rather, that it was confined to a subset (39%) of MVBs and that a second population of 6E10-negative MVBs existed (Fig. 1C). 6E10 staining was found on MVBs both sparsely and densely packed with ILVs, and so did not correlate with MVB maturation state. Lysosomes were not stained. 6E10 binds the first 16 residues of Aβ and thus detects full-length APP (fl-APP), the β C-terminal fragment (β-CTF) and Aβ. Similar results were obtained using another pan-Aβ antibody, 4G8 (data not shown). Several Aβ-specific antibodies tested on cell lines overexpressing APP showed no specific staining.

Western blotting H4-APP lysates using the 6E10 antibody showed the predominant species to be fl-APP with much less intense bands corresponding to the size of CTFs and little or no Aβ (Fig. 1D). These data suggest that, like many non-neuronal and neuronal cell lines, H4-APP cells produce very little Aβ and that the majority of the 6E10 signal detected by immunofluorescence and immunoelectron microscopy is likely to represent fl-APP. Consistent with this, treatment of H4-APP cells with the γ-secretase inhibitor DAPT, to prevent Aβ generation, caused no detectable change in 6E10 staining as assessed by immunoelectron microscopy (supplementary material Fig. S1A,B), and an antibody against the N-terminus of APP, which does not stain β-CTF or Aβ, showed the same dual localization to the Golgi and the ILVs of MVBs as 6E10 (supplementary material Fig. S1C,D).

**Fig. 2.**
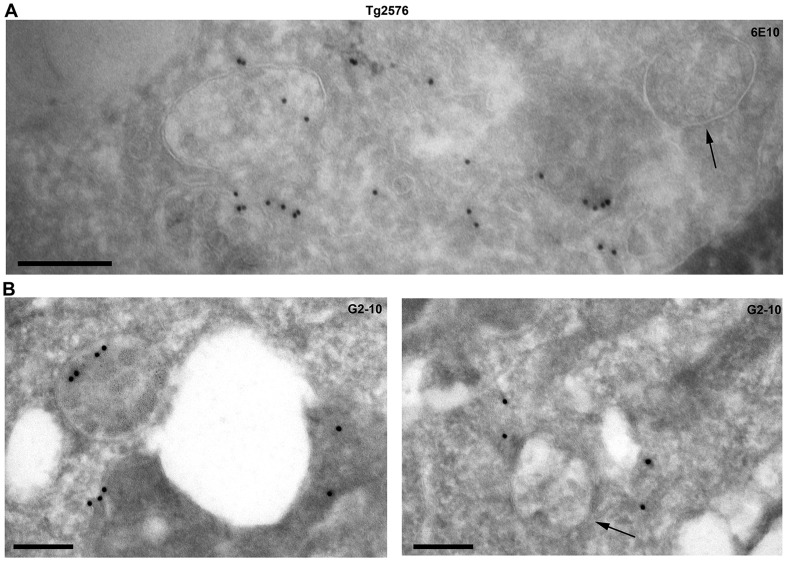
**APP and Aβ localizes to the ILVs of a subpopulation of MVBs in hippocampus of Tg2576 mice.** Ultrathin sections of hippocampus of Tg2576 mice were stained with the anti-APP/Aβ antibody 6E10 (A) or Aβ40-specific antibody G2-10 (B). Arrows show MVBs negative of gold labelling. Scale bars: 200 nm.

To determine whether Aβ could be detected in a subset of MVBs in neurons, hippocampus from age-matched wild-type and Tg2576 mice, which overexpress Swedish mutant APP (sweAPP) and present detectable levels of intraneuronal deposits of Aβ (Takahashi et al., 2002), were stained with anti-Aβ antibodies and assessed by immunoelectron microscopy. 6E10 and Aβ40-specific antibodies localized to the ILVs of a subset of MVBs in the Tg2576 mice (Fig. 2).

### APP traffics through EGFR-positive endosomes to lysosomes and is degraded

**Fig. 3.**
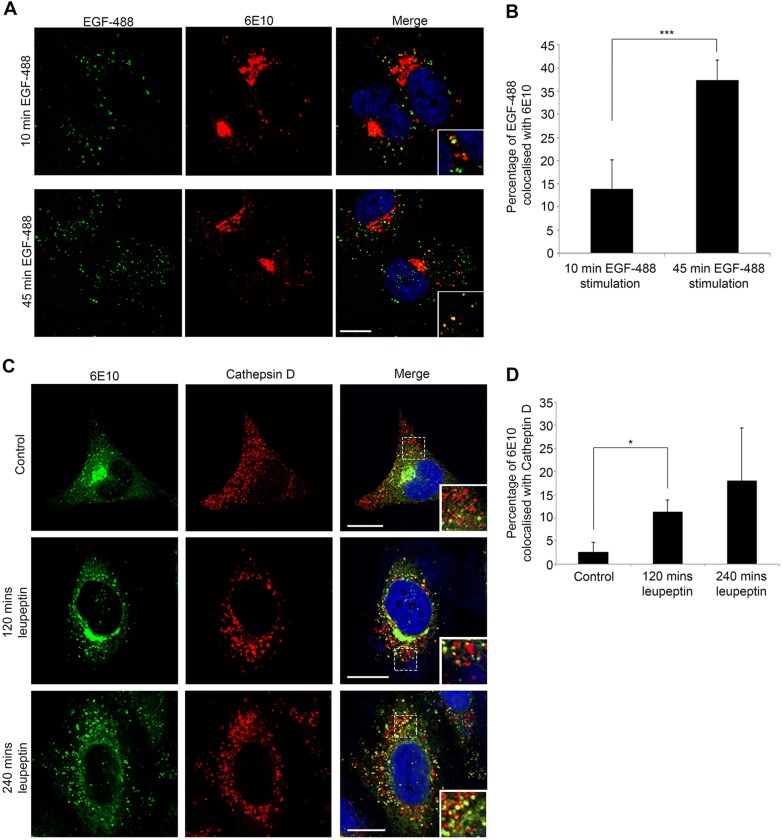
**APP traffics through the same MVBs as EGFR.** (A) H4-APP cells stimulated with EGF–Alexa-Fluor-488 (EGF-488; green) for 10 or 45 min were stained for APP/Aβ with 6E10 (red). Scale bar: 5 μm. (B) Quantification of APP colocalization with EGF-488. (C) H4-APP cells treated with leupeptin were stained with 6E10 (green), cathepsin D (red) and DAPI (blue). Scale bars: 10 μm. (D) Quantification of 6E10 colocalization with cathepsin D. All results are mean±s.d. (*n*=3 separate experiments). *P<0.05, ***P<0.005 (Student's *t*-test).

We have previously shown that EGFR traffics in a subpopulation of MVBs destined for the lysosome (White et al., 2006). To determine whether APP is trafficked in the same MVBs, cells were incubated with EGF-488 for 10 and 45 min when EGF–EGFR complexes are primarily localized to early endosomes and MVBs, respectively (Futter et al., 1996). Fig. 3A shows that a small proportion of punctate 6E10 staining colocalized with EGF after 10 min, suggesting that only a small proportion of 6E10-positive puncta are early endosomes (14% APP colocalized with EGF, Fig. 3B). However, after 45 min stimulation, the majority of 6E10-positive puncta contained EGF (37% of the total APP colocalized with EGF, the majority of the remainder was in the TGN). These data show that APP is trafficked in the same population of MVBs that deliver EGFR to the lysosome for degradation. Although in control cells, we did not detect APP in lysosomes (Fig. 1), treatment of H4-APP cells with the protease inhibitor leupeptin caused a time-dependent increase in the amount of APP within cathepsin-D-positive compartments (Fig. 3C,D). This indicates that APP can be trafficked to the lysosome but is rapidly degraded and only detectable following inhibition of lysosomal degradation.

### Depletion of Hrs inhibits traffic of APP to ILVs

**Fig. 4.**
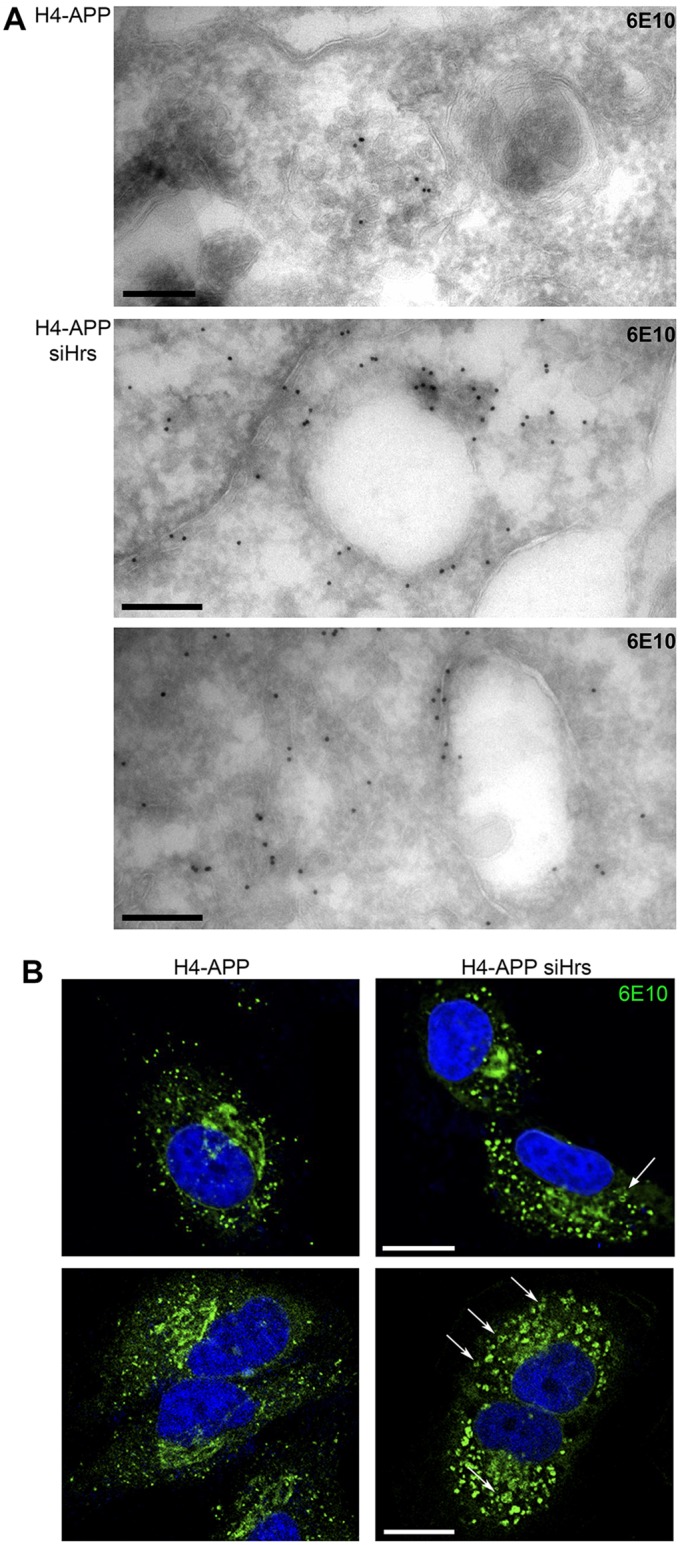
**Redistribution of APP from ILVs to the limiting membrane of MVBs after Hrs depletion.** (A) Cryosections of H4-APP and Hrs-depleted H4-APP cells (siHrs) were stained for APP/Aβ with 6E10 (upper two panels, cells fixed with 4% PFA; lower panel, cells fixed with 4% PFA and 0.2% glutaraldehyde). Scale bars: 200 nm. (B) Confocal microscopy reveals 6E10-positive endosomal rings following Hrs depletion (arrows). Scale bars: 5 μm.

Targeting of EGFR to the ILVs of MVBs depends on EGFR-ubiquitylation-dependent engagement of the ESCRT machinery. To measure potential ubiquitylation of APP N2a-UnT and N2a-APP cells were transfected with ubiquitin–Myc and lysates were immunoprecipitated with 6E10. Blotting of the immunoprecipitations with anti-Myc antibody revealed that fl-APP can be ubiquitylated (supplementary material Fig. S2A) and there are several potential sites of APP ubiquitylation (supplementary material Fig. S2B). Depletion of the ESCRT0 component Hrs (also known as Hgs), which binds ubiquitylated cargo, caused APP to redistribute from ILVs to the limiting membrane of enlarged endosomes, as shown by immunoelectron microscopy (Fig. 4A), such that 31.5% of 6E10 gold particles localized to the limiting membrane in control cells increasing to 73.6% in Hrs-depleted cells. Immunofluorescence analysis showed that the 6E10-positive punctae were clearly enlarged in Hrs-depleted cells (Fig. 4B) and sometimes 6E10-positive ‘rings' could be observed, as previously described (Choy et al., 2012). Some Hrs-depleted MVBs contained a population of unusually small ILVs, only detectable on glutaraldehyde-fixed specimens (Fig. 4A), as shown in our recent study on Hrs-depleted HeLa cells (Edgar et al., 2014).

### Hrs and Tsg101 depletion reduce lysosomal delivery of APP

**Fig. 5.**
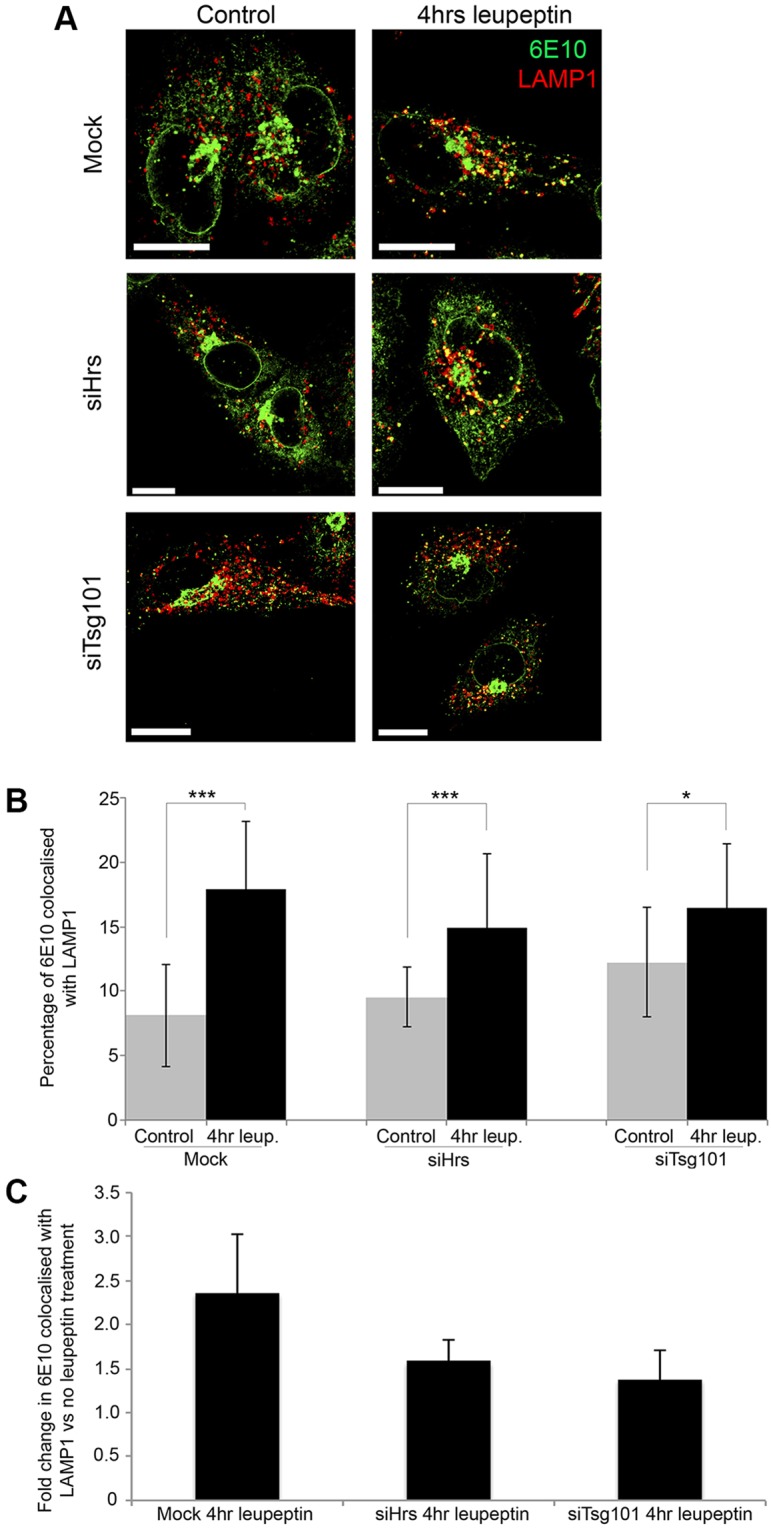
**Reduced lysosomal delivery after Hrs/Tsg101 depletion.** (A) H4-APP cells depleted of Hrs (siHrs) or Tsg101 (siTsg101) were treated with or without leupeptin and stained with 6E10 (green) and LAMP1 (red). Scale bars: 5 μm. (B) Quantification of 6E10 colocalization with LAMP1 (using Velocity). Results are mean±s.d. of three experiments. *P<0.05, ***P<0.005 (Student's *t*-test). (C) Fold change (mean±s.d., *n*=2 separate experiments) of 6E10 colocalized with LAMP1.

In order to determine whether depleting ESCRT components affected delivery of APP to lysosomes, we analysed the colocalization of APP with LAMP1. A change in the amount of co-staining of APP with LAMP1 upon ESCRT depletion could be caused by a change in the distribution of LAMP1 or by a change in the efficiency of delivery of APP to the lysosome. These two possibilities can be distinguished by incubation of the cells with leupeptin to inhibit lysosomal enzyme activity. This would be expected to have no effect on the distribution of LAMP1 but, as shown in Fig. 3, it increased APP signal in lysosomes by inhibiting degradation. Without leupeptin there was limited co-staining of APP with LAMP1 in control cells and cells treated with small interfering RNA (siRNA) against Hrs (siHrs) (∼8 and 9%, respectively) (Fig. 5A,B). With leupeptin there was a 2-fold increase in APP co-staining with LAMP1 in control cells compared with only a 1.5-fold increase in siHrs-treated cells (Fig. 5A,C), indicating inhibition of lysosomal delivery of APP in Hrs-depleted cells. Although cells treated with siRNA against Tsg101 (siTsg101) displayed higher APP and LAMP1 colocalization in controls, there was only a 1.25-fold increase following leupeptin treatment, indicating a greater inhibition of APP delivery to lysosomes in siTsg101-treated cells (Fig. 5A,C).

### Hrs or Tsg101 depletion reduces Aβ40 secretion but increases intracellular APP and Aβ

**Fig. 6.**
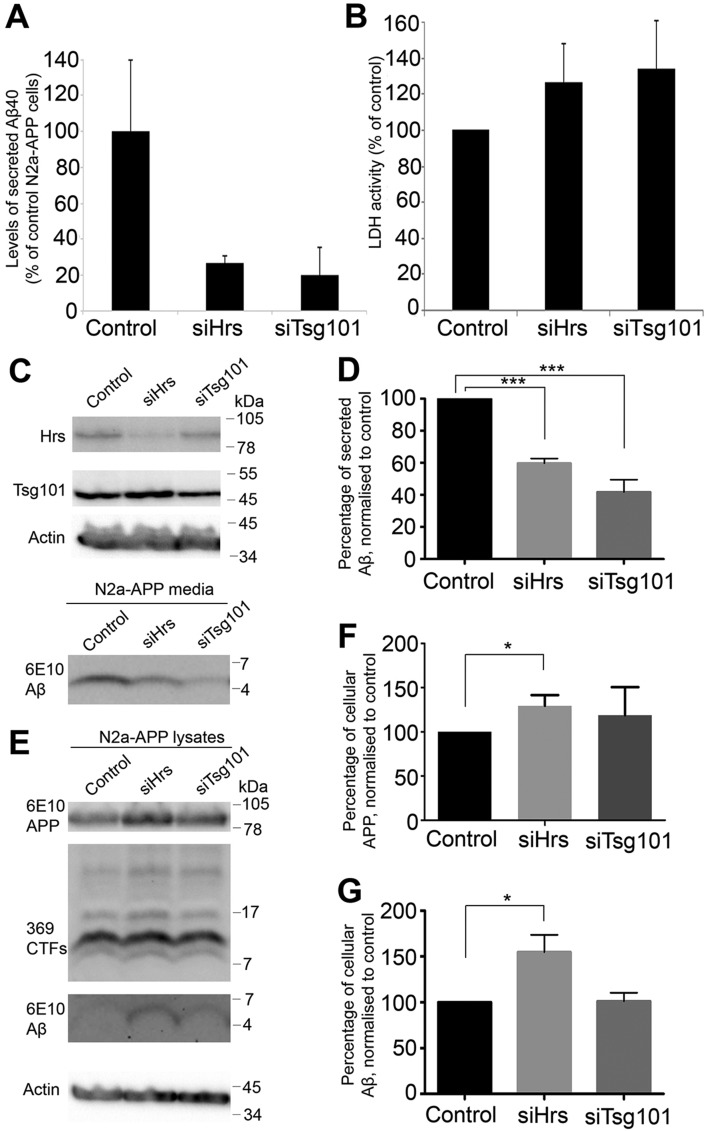
**Reduced Aβ40 secretion and increased intracellular APP and/or Aβ after depletion of Hrs or Tsg101.** (A) ELISA shows reduced Aβ40 secretion in N2a-APP cells depleted of of Hrs (siHrs) or Tsg101 (siTsg101). (B) Hrs or Tsg101 depletion does not affect release of LDH activity into culture medium. (C–G) N2a-APP cells were depleted of Hrs or Tsg101 and media (C,D) and cell lysates (E–G) were analysed by western blotting. All results are mean±s.d. of three experiments. *P<0.05, ***P<0.005 (Student's *t*-test).

The effects of ESCRT depletion on Aβ accumulation and secretion was tested on N2a-APP cells, which secrete more Aβ than H4-APP cells and show a similar redistribution of APP to the perimeter membrane of enlarged MVBs, frequently visible as ‘rings', upon Hrs or Tsg101 depletion (supplementary material Fig. S3). Depletion of Hrs or Tsg101 led to a 73% and 80% reduction in secreted Aβ40 levels respectively, as measured by ELISA of media samples (Fig. 6A), without any corresponding decrease in release of the intracellular enzyme lactate dehydrogenase (LDH) (Fig. 6B). Secreted Aβ levels do not necessarily reflect intracellular Aβ levels and so intracellular and secreted Aβ were analysed by western blotting, which, though challenging, allows distinction between fl-APP and processing products (Gouras et al., 2012). Western blotting of medium from N2a-APP cells showed reduced secreted Aβ levels following Hrs or Tsg101 depletion (Fig. 6C,D), in agreement with the ELISA results. Western blotting of cell lysates showed an increase in APP levels upon Hrs or Tsg101 depletion consistent with reduced delivery of APP to the lysosome (Fig. 6E,F). Interestingly, although α- and β-CTF levels were unchanged by Hrs or Tsg101 depletion (Fig. 6E), the intracellular levels of Aβ were dramatically increased following Hrs depletion (Fig. 6E,G). It is not possible to directly measure the effects of ESCRT depletion on Aβ generation, as the intracellular Aβ levels are a balance between production, degradation and secretion. However the intracellular Aβ accumulation that accompanied reduced Aβ secretion upon Hrs depletion suggests that Hrs-dependent targeting of APP to ILVs is not necessary for Aβ production but that Hrs and Tsg101 are involved in Aβ secretion.

## DISCUSSION

Despite the clinical importance of Aβ, the role of the endocytic pathway versus the TGN and the role of sorting within MVBs in regulating the intracellular accumulation and secretion of Aβ remain unclear. In keeping with previous studies, we found that APP localized predominantly to endosomes and the TGN, and now show that, within the endocytic pathway, APP is found mainly on the ILVs of a subset of MVBs. Sorting of APP onto ILVs could promote Aβ production by providing favourable conditions for amyloidogenic processing, as it does for PMEL in melanogenic cells. Consistent with this hypothesis, we found Aβ40 on the ILVs of MVBs in mouse brain from Tg2576 mice. Alternatively sorting of APP to ILVs could prevent intracellular Aβ accumulation by targeting APP and/or its processing products for lysosomal degradation. Consistent with this hypothesis, we found APP in EGFR-containing MVBs, an MVB subpopulation that normally fuses with lysosomes, and lysosomal accumulation of APP in cells treated with protease inhibitor.

To determine whether sorting of APP onto ILVs has a positive or negative effect on Aβ accumulation, we aimed to inhibit the ILV sorting machinery and analyse the effects on intracellular Aβ levels. EGFR is targeted to the ILVs of MVBs by ubiquitylation-dependent interaction with the ESCRT machinery. In contrast, PMEL, which depends on sorting to ILVs for amyloidogenic processing, is targeted to ILVs independently of ubiquitylation and the ESCRT machinery (Theos et al., 2006; van Niel et al., 2011). That APP traffics predominantly in the same MVBs that traffic EGFR implied that the ESCRT machinery was involved in APP sorting. However, ESCRT-dependent and -independent ILV sorting mechanisms are not entirely segregated within separate populations of MVBs. van Niel et al. (van Niel et al., 2011) have shown that the C-terminal fragment of PMEL that remains after amyloidogenic processing is targeted to the lysosome in an ESCRT-dependent manner and we have recently shown that Hrs-dependent and Hrs-independent ILVs can form in the same MVB (Edgar et al., 2014).

Several studies, including our own, show that APP can be ubiquitylated (Morel et al., 2013; Watanabe et al., 2012) and, hence, could potentially engage the ESCRT machinery. A previous study has shown that depletion of the ESCRT0 component Hrs or the ESCRTI component Tsg101 leads to the retention of APP in early endosomes at the expense of the TGN (Choy et al., 2012) and proposed a role for Hrs and Tsg101 in retromeric traffic of APP to the TGN. Another recent study presented evidence for a role for the ESCRT machinery in sorting APP to ILVs. Expression of a poorly ubiquitylated mutant APP or depletion of the phosphoinositide 3-kinase (PI3K) Vps34 and its effector Hrs inhibits sorting of APP to ILVs (Morel et al., 2013). Here, we show that depletion of Hrs or Tsg101 inhibit sorting of APP to ILVs of a subset of MVBs, directly demonstrating a role for the ESCRT machinery in sorting APP to ILVs.

Thus, two amyloidogenic proteins, PMEL and APP, are sorted onto ILVs by different mechanisms. ESCRT-independent sorting of PMEL to ILVs promotes amyloidogenic processing. Does sorting of APP onto ILVs, serve a similar purpose, albeit utilizing a different machinery, or does ESCRT-dependent ILV sorting target APP and its processing products to the lysosome and, thereby, reduce Aβ accumulation? Consistent with the latter possibility, we found that Hrs or Tsg101 depletion increased cellular APP levels and inhibited APP delivery to the lysosome. Morel et al. proposed that ESCRT-mediated ILV targeting limits Aβ production after finding that silencing Vps34, or expressing ubiquitylation-deficient APP, increased Aβ secretion (Morel et al., 2013). However, here, we found that Hrs or Tsg101 depletion reduced Aβ secretion. Choy et al. also found reduced Aβ secretion on Hrs or Tsg101 depletion but found increased Aβ secretion on depletion of the later ESCRT components CHMP and Vps34 (Choy et al., 2012). This enhanced Aβ secretion required retromer-dependent redistribution of APP to the TGN and these authors concluded that the TGN was the major site of Aβ production. How can treatments likely to reduce ESCRT-dependent sorting of APP to ILVs enhance Aβ secretion in one study (Morel et al., 2013) and reduce them in others (Choy et al., 2012) (and the current study)? Although differences in cell types might underlie these apparent differences (HEK293 cells in Choy et al. and Hela cells in Morel et al.), we can add a key component that was absent from previous studies. As well as Aβ secretion, we measured intracellular Aβ levels. Although depletion of Hrs inhibited Aβ secretion, intracellular Aβ levels were considerably increased. Although we cannot eliminate the possibility that Hrs depletion affects Aβ production, the elevated intracellular Aβ levels imply that Hrs-dependent sorting to ILVs does not promote Aβ generation. Aβ found on ILVs could arise from Aβ generation on the perimeter membrane of the MVB and subsequent sorting onto ILVs, or γ-cleavage could occur on ILVs as well as on the perimeter membrane. The previous immunoelectron microscopy findings showing that Aβ42 in neurons normally localizes preferentially to the MVB limiting membranes is consistent with Aβ generation on the MVB perimeter membrane (Takahashi et al., 2002). Intracellular Aβ levels depend on the balance between Aβ generation and Aβ degradation and secretion. Hrs or Tsg101 depletion might increase Aβ levels by inhibiting lysosomal delivery of APP or β-CTF, increasing the time that they reside in MVBs, but also increasing Aβ accumulation through reduced Aβ secretion.

What is the role of Hrs and Tsg101 in Aβ secretion? Although the major fate of ESCRT-dependent ILVs is degradation in the lysosome, Aβ accumulation in MVBs could affect that fate. Consistently, a defect in EGFR degradation has been observed upon Aβ accumulation within MVBs (Almeida et al., 2006). One alternative MVB fate is fusion with the cell surface and release of ILVs as exosomes, and exosome-associated Aβ has been found (Rajendran et al., 2006). Interestingly, one exosome population has been shown to be generated independently of ESCRTs (Trajkovic et al., 2008), but depletion of Hrs and Tsg101 has recently been shown to reduce some exosome production (Colombo et al., 2013; Tamai et al., 2012, 2010), and ubiquitylated proteins have been found in exosomes (Buschow et al., 2005). Thus, reduced exosome secretion could contribute to the reduced Aβ secretion upon depletion of Hrs or Tsg101. Additionally, non-exosome-associated Aβ within the MVB lumen could be released upon MVB fusion with the plasma membrane and Hrs or Tsg101 could play a role in this fusion. Another possible fate of MVBs is fusion with autophagosomes in a process that participates in autophagic degradation (Lamb et al., 2013). The accumulation of autophagic vacuoles in Alzheimer's disease brain (Nixon et al., 2005) and mouse Alzheimer's disease models (Yu et al., 2005) suggests a link between Aβ accumulation and autophagy. ESCRT proteins are required for efficient autophagic degradation (Rusten and Simonsen, 2008), but, in an intriguing recent development, genetic ablation of autophagy by knockout of Atg7 has been shown to dramatically reduce Aβ secretion (Nilsson et al., 2013; Nilsson and Saido, 2014). Clearly the relationship between ESCRT-mediated sorting of APP onto ILVs, autophagy and Aβ secretion is a major subject for future study.

Understanding the molecular mechanisms that regulate intracellular Aβ accumulation is crucial to understanding early Alzheimer's disease pathogenesis. Increasing evidence indicates that intracellular Aβ accumulation in Alzheimer's-disease-vulnerable neurons initiates synaptic dysfunction prior to the presence of amyloid plaques. Our data indicate a key role for early ESCRT components in limiting intracellular Aβ accumulation by sorting APP onto ILVs of MVBs for lysosomal degradation and promoting Aβ secretion.

## MATERIALS AND METHODS

### Reagents

Anti-APP/Aβ antibodies were mouse 6E10 and 4G8, against residues 3–8 and 17–24 of Aβ respectively (Covance), rabbit 369 against CTF (Buxbaum et al., 1990), and mouse G2-10 (Aβ40-specific) and P2-1 (N-terminus) (Millipore). Sheep anti-TGN46 (AbD Serotec), rabbit anti-Cathepsin D (Upstate), -LAMP1 (Ab24170 - Abcam), -Myc (Abcam), and mouse anti-Hrs (Enzo Lifesciences) and Tsg101 (Genetex) antibodies were used. Leupeptin and cycloheximide (Sigma) were used at 50 μg/ml and 40 μg/ml, respectively.

### Cell culture

Human H4 neuroglioma cells (H4) cells from ATCC were cultured in Dulbecco's modified Eagle's medium (DMEM) with 10% fetal calf serum (FCS). N2a mouse neuroblastoma cells (generously provided by Gopal Thinakaran and Sangram Sisodia – University of Chicago) were cultured in 47.5% DMEM, 47.5% OptiMem and 5% FCS, with 0.4 mg/ml Geneticin (Invitrogen).

### Western blotting

Western blotting to analyse knockdown efficiencies were performed as previously described (Edgar et al., 2014). To perform western blotting for Aβ, medium was collected and cells were harvested in ice-cold PBS. Cell pellets were lysed in 6% SDS, 1% β-mercaptoethanol, sonicated and heated at 95°C. Centrifuged (10,600 ***g*** for 10 min) supernatants were electrophoresed, transferred to PVDF membranes (Millipore), and boiled in PBS. Blocking and antibody incubations were in 0.1% Tween-20 (PBST) with 5% milk powder. Immunoreactions were visualized by chemiluminescence (Pierce) and quantified with Image Lab (Bio-Rad).

### Co-immunoprecipitation

The ubiquitin–Myc construct was a kind gift from Sylvie Urbe (University of Liverpool, UK). Cells were scraped in lysis buffer containing 10 mM NEM and lysates incubated with 10 μg of primary antibody for 4 h and then protein G beads (Santa Cruz Biotechnology) for 1 h. Washed pellets were boiled in reducing sample buffer and analysed by SDS-PAGE and western blotting.

### RNA interference

Cells were transfected using Oligofectamine (Invitrogen) with 20 μM negative control siRNA (Mammalian AllStar negative siRNA), or human Hrs or Tsg101 (Razi and Futter, 2006), murine Hrs ON-TARGETplus SMARTpool siRNA or murine Tsg101 ON-TARGETplus SMARTpool siRNA sequences (Quiagen).

### Cryo-immunoelectron microscopy

Cultured cells were prepared and sectioned as described (Edgar et al., 2014). For mouse brains, 10-month-old mice expressing human-sweAPP (Tg2576 mice; Hsiao et al., 1996) and wild-type littermates were perfusion fixed with 4% PFA. 50-μm vibratome sections of hippocampus were dissected and embedded in 12% gelatin before preparation as above. Sections were immunogold labelled (Slot et al., 1991) and visualized with a JEOL1010 transmission electron microscope with a Gatan digital camera and Digital Micrograph software. All animal experiments were approved by the Animal Ethical Committee of Lund University (Sweden).

### Immunofluorescence

Cells were fixed with 4% PFA, permeabilized with 0.1% saponin, and then blocking, antibody incubations and washes were in 1% BSA and 0.01% saponin in PBS. Coverslips were mounted in DAPI-containing medium Prolong gold anti-fade reagent (Invitrogen). For EGF-trafficking experiments, cells were serum starved for 90 min before incubation with 200 ng/ml EGF–Alexa-Fluor-488 (Molecular Probes).Cells were visualized using a Leica TCS SP2 AOBS confocal microscope using a 63× oil immersion lens. Images were analysed using Methamorph Image Analysis or Velocity software.

### Human Aβ40 ELISA

Human Aβ40 secreted over 48 h was measured in culture medium by ELISA (Invitrogen).

## Supplementary Material

Supplementary Material
