# Supplementary material for: ESCRTs regulate amyloid precursor protein sorting in multivesicular bodies and intracellular amyloid-β accumulation
Source: J Cell Sci. 2015 Jul 15;128(14):2520–8. doi: 10.1242/jcs.170233 (PMC4510853; doi:10.1242/jcs.170233)
Supplement: Supplementary Material [file supp_128_14_2520__index.html]

Supplementary Material 

# ESCRTs regulate amyloid precursor protein sorting in multivesicular bodies and intracellular amyloid-β accumulation

## JCS170233 Supplementary Material

- Supplementary Material
