# Supplementary material for: ESCRTs regulate amyloid precursor protein sorting in multivesicular bodies and intracellular amyloid-β accumulation
Source: J Cell Sci. 2015 Jul 15;128(14):2520–8. doi: 10.1242/jcs.170233 (PMC4510853; doi:10.1242/jcs.170233)
Supplement: Supplementary Material [file supp_jcs.170233_JCS170233supp.pdf]

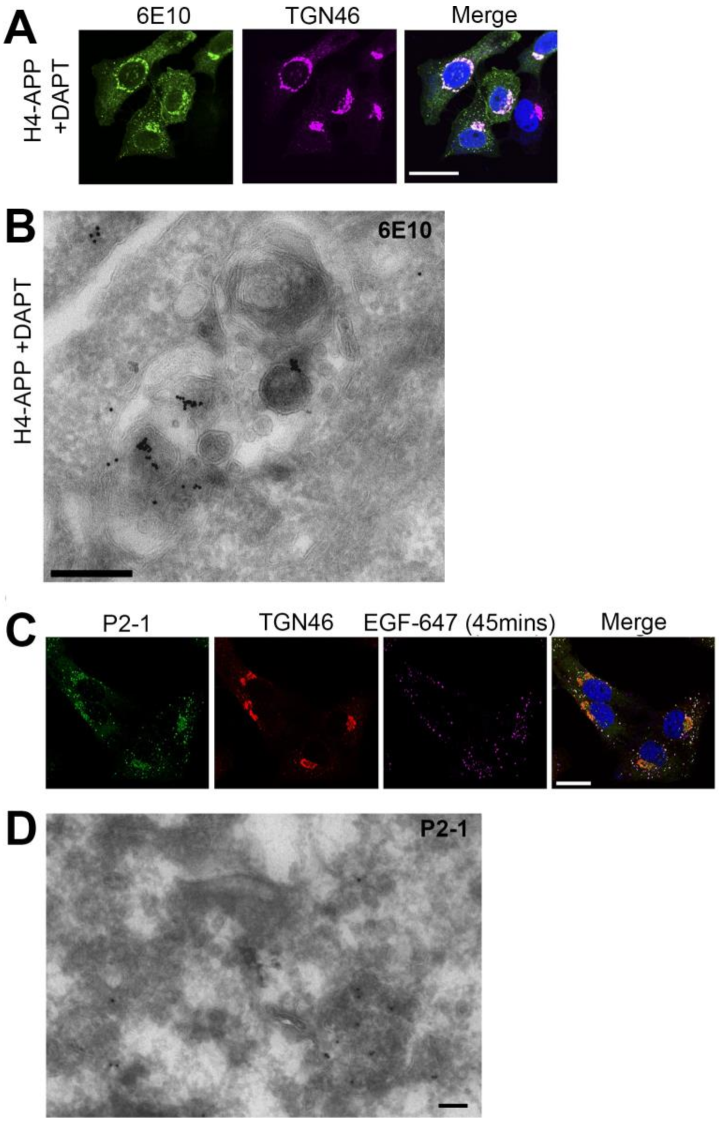

Supplementary Figure 1 – Inhibition of y-secretease does not alter 6E10, and the N-terminus of APP displays the same localization at 6E10. (A) H4-APP cells were treated with the y-secretase inhibitor DAPT (1 ng/ml, 18 hours) before being fixed and analysed by immunofluorescence. Cells were stained with the anti-APP/Aβ antibody 6E10 (green) and an anti-TGN46 antibody (magenta). Scale bar 10µm. (B) Cells were similarly treated with DAPT and processed for cryosectioning. Thawed cryosections were stained with the anti-APP/Aβ antibody 6E10. Scale bar 200nm. (C) H4-APP or untransfected cells were fixed and stained with the N-terminal APP antibody, P2-1 (green) and imaged by confocal microscopy. Scale bar 10µm. (D) H4-APP cells were fixed and prepared for cryosectioning. Ultrathin cryosections were stained using the anti-N-terminal APP antibody, P2-1. Scale bars 100nm.

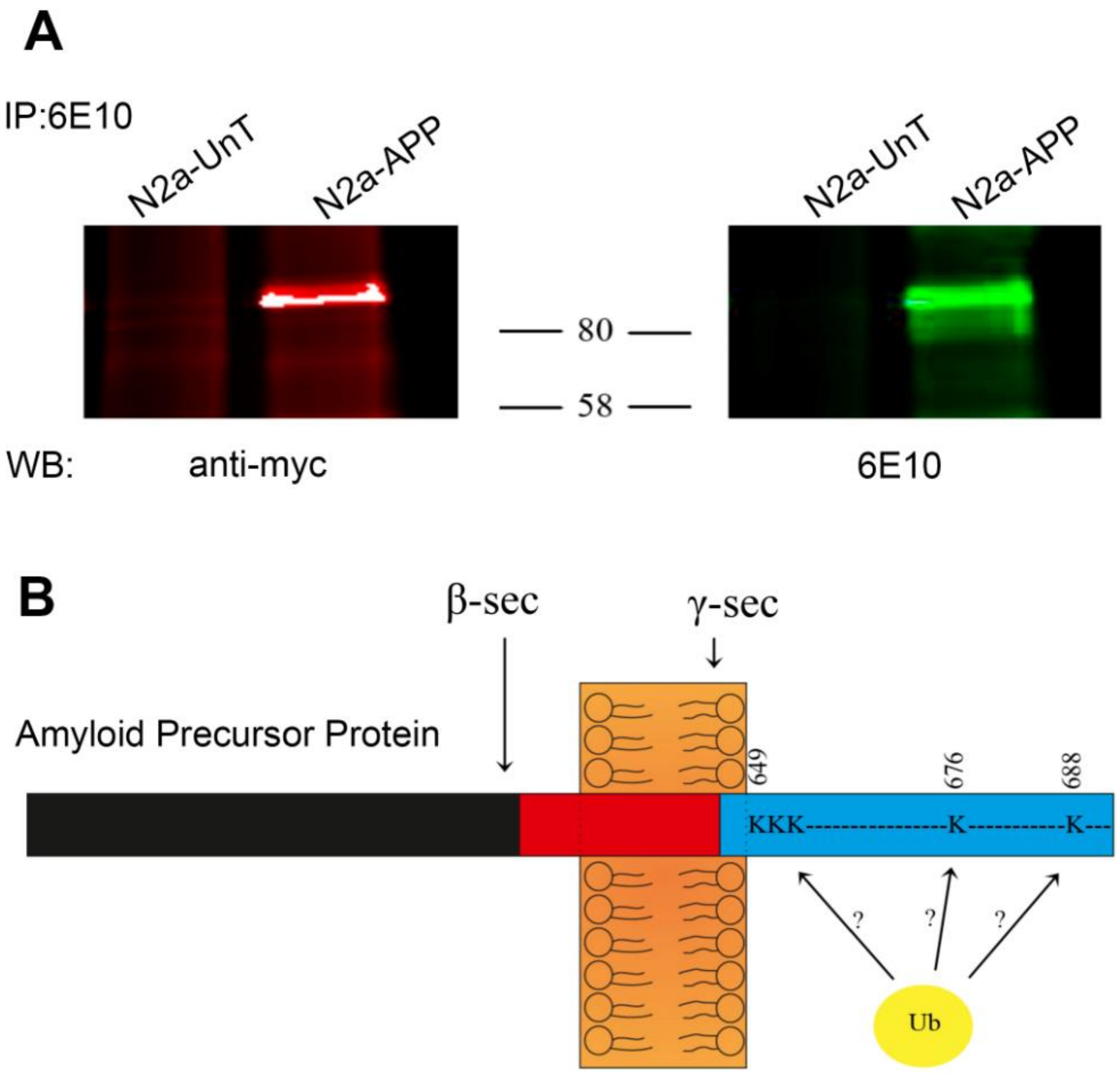

Supplementary Figure 2 – APP undergoes ubiquitination. (A) N2a-UnT and N2a-APP cells transfected with a myc-tagged ubiquitin construct were lysed and immunoprecipitated using anti-APP/Aβ antibody, 6E10. Pull-downs were Western blotted with anti-myc antibody. (B) Schematic of potential sites of ubiquitination within APP.

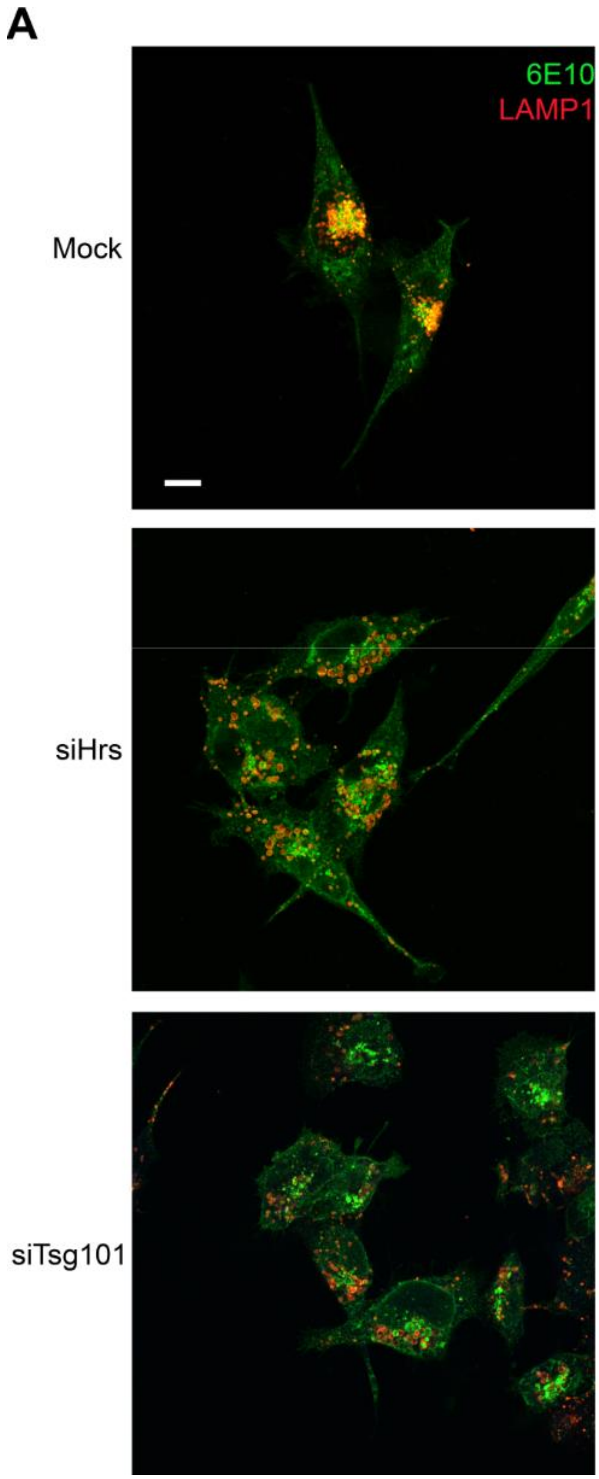

Supplementary Figure 3 - N2a-APP cells display similar morphology to H4-APP cells following siHrs or siTsg101 depletion. N2a-APP cells were depleted for Hrs or Tsg101 and stained for APP (green) and LAMP1 (red). Scale bar 10μm.
